# Impact of Community-Based DOT on Tuberculosis Treatment Outcomes: A Systematic Review and Meta-Analysis

**DOI:** 10.1371/journal.pone.0147744

**Published:** 2016-02-05

**Authors:** HaiYang Zhang, John Ehiri, Huan Yang, Shenglan Tang, Ying Li

**Affiliations:** 1 College of Preventive Medicine, Third Military Medical University, Chongqing, China; 2 Department of Health Promotion Sciences, Mel & Enid Zuckerman College of Public Health, University of Arizona, Tucson, Arizona, United States of America; 3 Department of Hygienic Toxicology, Key Lab of Medical Protection for Electromagnetic Radiation, Ministry of Education of China, Third Military Medical University, China; 4 Duke Global Health Institute, Duke University, Durham, North Carolina, United States of America; 5 Department of Social Medicine and Health Service Management, College of Preventive Medicine, Third Military Medical University, Chongqing, China; University of Ottawa, CANADA

## Abstract

**Background:**

Poor adherence to tuberculosis (TB) treatment can lead to prolonged infectivity and poor treatment outcomes. Directly observed treatment (DOT) seeks to improve adherence to TB treatment by observing patients while they take their anti-TB medication. Although community-based DOT (CB-DOT) programs have been widely studied and promoted, their effectiveness has been inconsistent. The aim of this study was to critical appraise and summarize evidence of the effects of CB-DOT on TB treatment outcomes.

**Methods:**

Studies published up to the end of February 2015 were identified from three major international literature databases: Medline/PubMed, EBSCO, and EMBASE. Unpublished data from the grey literature were identified through Google and Google Scholar searches.

**Results:**

Seventeen studies involving 12,839 pulmonary TB patients (PTB) in eight randomized controlled trials (RCTs) and nine cohort studies from 12 countries met the criteria for inclusion in this review and 14 studies were included in meta-analysis. Compared with clinic-based DOT, pooled results of RCTs for all PTB cases (including smear-negative or -positive, new or retreated TB cases) and smear-positive PTB cases indicated that CB-DOT promoted successful treatment [pooled RRs (95%CIs): 1.11 (1.02–1.19) for all PTB cases and 1.11 (1.02–1.19) for smear-positive PTB cases], and completed treatment [pooled RRs (95%CIs): 1.74(1.05, 2.90) for all PTB cases and 2.22(1.16, 4.23) for smear-positive PTB cases], reduced death [pooled RRs (95%CIs): 0.44 (0.26–0.72) for all PTB cases and 0.39 (0.23–0.66) for smear-positive PTB cases], and transfer out [pooled RRs (95%CIs): 0.37 (0.23–0.61) for all PTB cases and 0.42 (0.25–0.70) for smear-positive PTB cases]. Pooled results of all studies (RCTs and cohort studies) with all PTB cases demonstrated that CB-DOT promoted successful treatment [pooled RR (95%CI): 1.13 (1.03–1.24)] and curative treatment [pooled RR (95%CI): 1.24 (1.04–1.48)] compared with self-administered treatment.

**Conclusions:**

CB-DOT did improved TB treatment outcomes according to the pooled results of included studies in this review. Studies on strategies for implementation of patient-centered and community-centered CB-DOT deserve further attention.

## Introduction

Despite the overall global decline in tuberculosis (TB) incidence and mortality, the disease remains the second leading cause of death from infectious diseases globally, just after HIV/AIDS [1]. Global tuberculosis report in 2014 estimated that there were 9.0 million TB cases and 1.5 million TB mortalities in 2013 [1]. The emergence of multidrug-resistant (MDR) TB and extensively drug-resistant TB has further heightened the public health importance of TB control efforts. There is concern that the estimated 2012 global incident cases of MDR-TB of 450,000 and mortality of 170,000 may only represent about one-third of actual cases of MDR-TB [1]. Drug resistance primarily arises from poor treatment adherence or incorrect drug usage. Early diagnosis of patients and rapid initiation of effective therapy are essential in the prevention of MDR-TB [2].

The effective TB control policy recommended by the World Health Organization (WHO) is Directly Observed Therapy, Short-course (DOTS) [3]. Directly observed treatment (DOT) is the key component of DOTS [3]. DOTS supervisors are required to encourage and observe patients swallow their anti-TB drugs during the course of their treatment [4]. A DOT supervisor can be a clinical staff member, an employer, a teacher, a family member, or a lay volunteer who may be professional or amateur [5]. DOT can be beneficial for prevention, diagnosis, support, and care [1], with its primary aim being an improvement in medication adherence [6]. Decentralization of treatment expands access to healthcare services for all stakeholders, increases access to effective TB treatment [7,8], and results in more equitable provision of available treatment [9]. The purpose of decentralizing TB services, including community TB care is to increase access to DOTS and to improve program performance, especially in relation to detection of new smear-positive cases and treatment success rates [10]. Evidence also shows that decentralization of TB services does not compromise treatment outcomes [11]. Community-based TB care refers to a decentralized program of TB services that is implemented in settings where patients live, work, and receive education [12]. As a component of community-based TB care, Community-based DOT (CB-DOT) is designed to relieve the pressure of patient care on over-stretched health facilities in countries with a high TB burden [13]. Patients can remain in their homes, at their workplaces, or schools during therapy rather than traveling long distances and waiting for long hours in healthcare facilities [14]. This is particularly important in areas with poor access to health services. For such settings, care in the community can have a significant impact on improving treatment outcomes [1].

Many studies have investigated the effects of CB-DOT. However, the results of these studies have been inconsistent. For example, some studies reported a higher treatment success rate in TB patients treated under CB-DOT [4, 15] compared with those treated under clinic-based DOT, while other studies [16–18] reported no significant difference. A randomized controlled trial by Lwilla et al [17] reported that fewer patients died during treatment under CB-DOT than under institution-based DOT, while another study [19] found no differences in mortality rates between CB-DOT and clinic/self-administered TB treatment groups. There is a need to critically review and appraise current evidence in order to elucidate the effect of CB-DOT on TB treatment. One meta-analysis investigated the impact of lay health workers (LHWs). However, the study did not specifically focus on DOT by community members who did not have a healthcare background, and did not explore the impact of CB-DOT on improving negative treatment outcomes (treatment default, death, treatment failure, and transfer out) [20]. A review by Volmink and Garner [21] analyzed the differences in cure rate and successful treatment rate (cure or completion) by comparing different approaches to treatment: self-administered treatment and home-based DOT compared with clinic-based DOT; clinic-based DOT compared with DOT by a family member or community health worker; and DOT by a family member compared with DOT by a community health worker. The review did not compare DOT by community volunteers with clinic-based DOT, DOT by family member or workplace DOT, and did not elucidate the effect of CB-DOT on negative outcomes. A systematic review and meta-analysis published in 2015 by Karumbi and Garner [22] compared the differences of treatment outcome between DOT and self-administered treatment, DOT at home and DOT at health facility, DOT by family member and DOT by a community health worker. But this review also only focused on outcomes related to cure or treatment completion and did not explore the impact of CB-DOT on improving negative treatment outcomes (treatment default, death, treatment failure, and transfer out). Another by Munro et al. [23] compared the effect of DOT and Self-Administered Therapy (SAT) in preventing microbiologic failure, relapse, or Adverse Drug Reaction (ADR), but did not compare DOT by different volunteer community members.

To address these gaps in knowledge, we examined the effect of CB-DOT on both positive treatment outcomes (cured treatment, completed treatment and successful treatment) and negative treatment outcomes (default, death, failure treatment, transfer out and interrupted treatment rate) compared with clinic-based DOT, family-based DOT, workplace-based DOT, and self-administered treatment by reviewing all available randomized controlled trials (RCTs) and cohort studies.

## Methods

### Search strategy

This review was performed according to the standard procedures of the Cochrane Collaboration [24] and the Preferred Reporting Items for Systematic Reviews and Meta-Analyses (PRISMA) checklist (S1 PRISMA Checklist) [25]. To identify eligible prospective studies published in English up to February 2015, we searched Medline/PubMed, EMBASE, and EBSCO (PsycINFO and CINAHL). A mix of free text and index terms were used to maximize the retrieval of potentially relevant studies (S1 Text). We sought unpublished data from the grey literature through Google and Google Scholar. We hand-searched reference lists of identified articles.

### Inclusion/exclusion criteria

The following criteria were employed:

1. *Types of studies*: Follow-up studies including randomized controlled trials (RCTs) and prospective cohort studies were included.
2. *Participants*: Patients with pulmonary TB (PTB) (including smear-positive or -negative PTB, newly diagnosed cases, and those undergoing retreatment were included. For studies that reported treatment outcomes of participants with smear-positive TB and smear-negative TB, and new or retreated TB and ex-pulmonary TB patients separately, [4,16,26,27], we only included results of TB patients without ex-pulmonary TB. Studies were excluded where we could not separate PTB patients from extra-PTB patients [28–30].
3. *Type of interventions*: Studies in which CB-DOT services were provided by lay/community health workers, volunteers, peers, friends, etc. [31–33]. For the purpose of this review, we defined CB-DOT as DOT that was delivered by lay healthcare personnel (including village health workers)/community health workers or voluntary lay individuals from the community (not including family or workplace individuals).
4. *Outcomes measures*: We used the following definitions of treatment outcomes for non MDR-TB [1,34]: (i) Successful treatment: a patient who was cured or who completed treatment; (ii) Cured treatment: a patient who was initially sputum smear-positive and who was sputum smear-negative in the last month of treatment and on at least one previous occasion; (iii) Completed treatment: a patient with sputum smear-positive or sputum smear-negative pulmonary TB who completed treatment; (iv) death: a patient who died from any cause during treatment; (v) Failure: a patient who was initially sputum smear-positive and who remained sputum smear-positive at month 5, or was later found to have a MDR strain at any point during treatment, whether they were smear-negative or smear-positive; (vi) Default: a patient whose treatment was interrupted for two consecutive months or more; and (vii) Transfer out: a patient who was transferred to another reporting unit and whose treatment outcome was unknown.

When there was evidence of multiple publications of the same study over time, only the article with a full report was included.

### Study selection

Two reviewers (HZ and YL) used the above inclusion and exclusion criteria to identify relevant studies. Each reviewer independently screened the titles and abstracts of identified studies to assess their eligibility for inclusion in the review, using an eligibility form based on the inclusion criteria. Where there was disagreement, a decision to include a study was reached after discussion and consensus by among all reviewers.

### Quality assessment

Two reviewers (HZ and YL) independently assessed the methodological quality of included studies. For RCTs, we assessed generation of the allocation sequence and concealment of allocation as adequate, inadequate, or unclear [35]. Blinding was classified as adequate if steps were taken to ensure that those recording the main outcome of the study were blind to the assigned interventions, and inadequate if this was not the case, or if there was no description of the method for assessing the adequacy of the randomization procedure. Completeness of follow-up was assessed as adequate if it included 90% or more, inadequate if it included less than 90%, or unclear if it was not mentioned.

We assessed the quality of cohort studies using the Newcastle-Ottawa Scale [36]. For cohort studies, we assessed the representativeness of the exposed cohort in the study setting, the selection of a non-exposed cohort, the ascertainment of exposure, demonstration that the outcome of interest was not present at initiation of the study, comparability of the cohorts on the basis of study design and analyses, outcome assessment, and the adequacy of follow-up [36]. The assessment is presented using a scoring system, where 1 indicates that the study met the criteria; 0 indicates the study did not meet the criteria; ND indicates that fulfilment of the criteria could not be determined. After assessing the quality of each included study on the basis of these criteria, a composite quality score was assigned, ranging from 0 to 9. Studies that scored ≥ 8 were judged to be of high quality.

### Data abstraction

Data from eligible studies were independently abstracted by two reviewers (HZ and YL). Differences were resolved by discussion and consensus among all reviewers. Data extracted from each study included: name of first author/year of publication, type of study design, place of study, type of participant (newly diagnosed/retreatment and smear-positive/negative pulmonary TB patients) age, type of intervention (comparison groups and sample size of each group), outcomes (successful treatment, completed treatment, cured treatment, death, default, failure, and transfer out), and the main results of each study.

### Assessment of heterogeneity

Heterogeneity between studies was evaluated using the Q test [37] and the I-squared statistic (I^2^ = 100% × (Q-df)/Q) [38]. For the Q test, a p-value ≤ 0.10 was considered to indicate significant heterogeneity among the studies. Where the p-value was ≤ 0.10, we calculated I^2^, and studies with I^2^ ≤ 50% were deemed acceptable for inclusion in the meta-analysis. Where heterogeneity was significant, subgroup analysis was conducted to explore possible reasons for the heterogeneity. In the subgroup analyses, the heterogeneity within groups was also tested, using the same statistical methods.

### Data Synthesis

The first step in data analysis involved a synthesis aimed at summarizing, comparing, and contrasting the extracted data. Meta-analyses were then conducted to assess the impact of CB-DOT on seven treatment outcomes compared with clinic-based DOT, with family-based DOT (DOT provided by family members), workplace-based DOT (DOT provided by workplace individuals), and self-administered treatment. Pooled risk ratio (RR) with 95% confidence interval (CI) was calculated separately by outcome and compared using RevMan 5.2 (Cochrane Collaboration) [24]. The fixed effects model was used to pool studies where the level of heterogeneity was acceptable (i.e., p > 0.10 or p ≤ 0.10, but I^2^ < 50%), and the random effects model, where significant heterogeneity was found between studies (p ≤ 0.10, I^2^ > 50%). Subgroup analysis was also conducted to explore factors associated with heterogeneity.

## Results

### Description of studies

**Fig 1:**
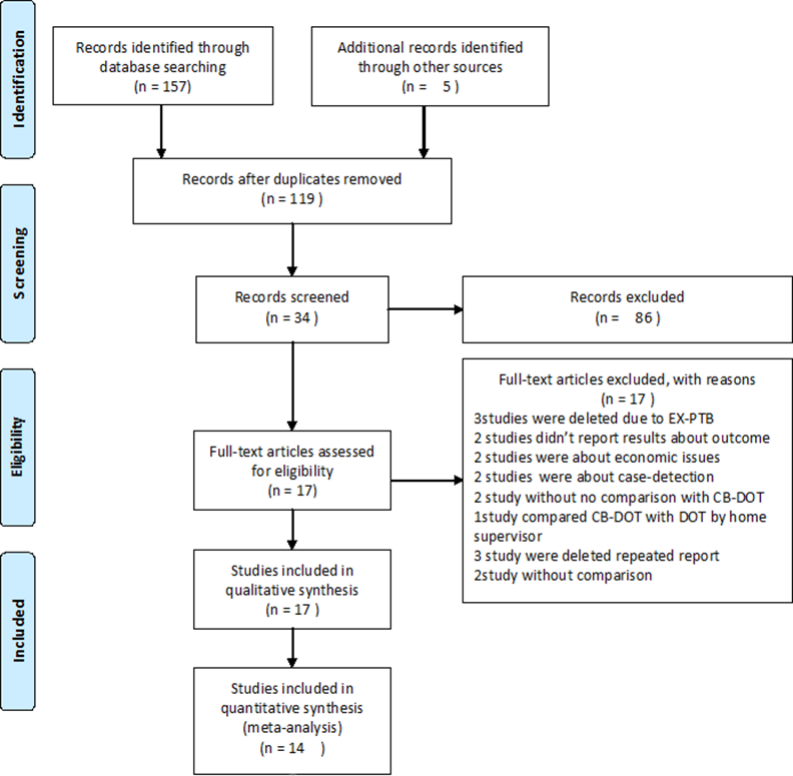
Results of literature search. This figure is a description of the full search process.

Fig 1 presents an illustration of the search output. The initial search yielded 119 potentially relevant articles. After reviewing the titles, abstracts, and full texts, 17 studies involving 12,839 pulmonary TB patients from 12 countries (Table 1) met the criteria for inclusion in the review. Ten studies were conducted in Africa [15–17,19,26,39–42], five of which were in South Africa [15,39–42]; seven [18,31,43–46] were from countries in Asia, and one [27] was in South America. Eight of the 17 studies [15–18,31,42,45] were RCTs and nine were prospective cohort studies. Eight studies [15–16,26–27,31,43,45] included only new smear-positive PTB cases; four studies [17–19,46] included all smear-positive PTB cases; one study [46] included both smear-positive and -negative PTB cases; and two studies [39,42] had no description about smear-positive or–negative cases. All 17 studies were included in the systematic review. All eight RCTs [15–18,31,42,45] with 3,456 PTB patients, and six cohort studies [26,39–41,43,44] with 3,456 TB patients had adequate data for inclusion in the meta-analysis.

**Table 1:** General information for included studies for Community Based intervention and Treatment (17studies).

Studies	Design	Region	Population (Age, type of TB)	Type and period of DOT and sampling size	Additional measures to promote treatment	Outcome	Main results
Wright, 2004[4]	RCT	Swaziland	Adult and children patients, new SS+ PTB	DOT by CHWs; Entire standard course of treatment; 290 cases with DOTS by CHWs /296 cases with DOT by Family member	Both CHWs and family members reminded them if they forgot and recorded adherence on a Treatment Supporter Card. Defaulters were notified to the diagnostic centre.	Completed treatment, cured treatment, death, failure, default, transferred out	The overall cure rate was 65% among new SS^+^ PTB cases; this increased to 70% when cure and treatment completed rates were combined. There was a non-significant difference in cure rate between the two groups [7% difference (0–15%), exact P = 0.10]. The difference between groups when cure and treatment-completed rates were combined approached significance [7% difference (0–15%), exact P = 0.06]. Death: 11% CHWs DOT vs.18% family member DOT Failure: 0% CHWs DOT vs.0% family member DOT; Default: 12% CHWs DOT vs.13% family member DOT; Transfer out: 3% CHWs DOT vs.3% family member DOT
Clarke, 2005[15]	Cluster RCT	South Africa	≥15 years patients, new SS^+^ PTB	DOT by LHWs (trained farmers or LHWs played a mentoring role; Entire standard course of treatment;75 cases in intervention group /89 cases in control	Visiting the patient and encouraging and monitoring treatment adherence regularly for self-supervision	Successful treatment failure, interrupted treatment, transfer out, death	The successful treatment completion rate in NSP adult TB patients was 18.7% higher [95%CI (0.9, 36.4)] on farms in the intervention group than on farms in the control group. There were no significant differences on the rest treatment outcomes.Failure: 7% LHW DOT vs.3% Clinic DOTInterrupted treatment: 8% LHW DOTS vs.16% Clinic DOT; Transfer out: 3% LHW DOT vs.3% Clinic DOT;Death:1% LHW DOT vs.3% Clinic DOT
Wandwalo, 2004[16]	Un-blinded RCT	Tanzania	≥5 years patients, new SS+ PTB	CB-DOT by guardians and former TB patients; 2 months of intensive treatment phase; 143 cases with CB-DOT /179 cases with health facility-based DOT	Recording drug taking, encouraging patients during treatment.	Successful treatment, cured treatment, Completed treatment, death, failure, default, transferred out	Treatment success rates were 85% and 83% among patients under CB-DOT and health facility-based DOT, respectively [OR (95% CI): 1.17(0.75, 1.83)]. There were no significant differences on the rest treatment outcomes. The cure rate: 78% CB-DOT vs.79% health facility-based DOT. Completed treatment: 9.8% CB-DOT/3.4% health facility DOT. Death: 7.0% CB-DOT vs.11.2% health facility DOT. Failure: 0% CB-DOT vs. 1.1% health facility DOT Default: .2.8% CB-DOT vs.3.4% health facility DOT Transferred out: 2.8% CB-DOT vs. 2.1% health facility DOT
Lwilla, 2003[17]	Cluster RCT	Tanzania	Patients with mean age(years) (SD): IBDOT 35.7(14.1) and CBDOT 35.5(13.3), SS^+^ PTB	CB-DOT by a community member, living in the same village as that of the patient; 2 months of intensive treatment phase; 221 cases with CB-DOT/301 cases with IB DOT	checking treatment card and counting pills during house visit; a sputum check follow-up	Cured treatment, transferred out, death, failure,	There was no significant difference in cure rates between the two strategies [M-H pooled OR (95%CI):1.58(0.32, 7.88)]. Fewer patients randomized to CBDOT died than under IBDOT (OR (95%CI):0.13(0.03, 0.65) at 2 months and 0.12(0.02, 0.89) at7 months]. Transfer out: 14.75% IBDOT vs. 4.98% CBDOT [OR (95%CI):0.29(0.07,1.28)]. Failure: 1.33% IBDOT vs. 5.29% CBDOT [OR(95%CI):0.63(0.13, 3.15)]
Kamolratanakul,1999[18]	RCT	Thailand	≥15 years old, SS^+^ PTB	CB DOT by village health volunteers or other community leaders; Entire standard course of treatment; 24 cases with DOTS by HW/ 34 DOT by community members /352 cases with DOTS by family members	Treatment monitoring card, home visit.	Successful treatment, completed treatment, default, failure, transfer out and death	No significant differences in outcomes could be observed between patient groups receiving DOT under the various options for treatment supervisors. Cure:79% Health workers vs.74% community members vs.77% family members Complete: 88%Health workers vs.79% community members VS. 84%family members. Death: 8%Health workers vs.3%community members/6% family members Failure: 0% Health workers vs.0% community members/2% family members Default:4% Health workers vs.15% community members /6% family members Transfer out: 0% Health workers vs.3% community members /2% family members
Zvavamwe, 2009[19]	Cohort study	Namibia	no description about ages of patients, SS^+^ PTB	Community-based TB treatment by family members of TB patients and other selected members of the community; Entire standard course of treatment; 308 cases with CB-DOT /24 cases with clinic-based DOT or self-administrated treatment	encouraging observing, documenting daily treatment and providing information about TB to the communities	Cured treatment and death	However, there was a statistically significant difference in cure rates between these two groups (x^2^ = 11.78, p≤0.05, and RR = 1.35, p = 0.05). There was no difference in death rate between community-based and the clinic/self-administered TB treatment groups (x^2^ = 3.01, p>0.05)
Cavalcante,2007 [26]	Longitudinal cohort study	Brazil	Adult patients, new SS^+^ PTB	CB DOT by CHWs who underwent a training program designed to teach them how to provide TB care in the community, focusing on TB control and DOT administration. Entire standard course of treatment; 489 cases with DOTS by CHWs /726 cases with clinic DOT/ 596 cases with SAT	Encouraging non-adherent patients to continue treatment, monthly clinical evaluation and microscopy	Successful treatment	Treatment success rates for new smear-positive and retreatment TB cases were significantly higher among those treated with CB-DOT compared to clinic-based DOT. 81.4% SAT vs. 84.6% Clinic-based DOT vs. 90.1% CB-DOT
Adatu, 2003[27]	Pre-post cohort study	Uganda	Patients with median age (years): pre-CB-DOTS 32 and CB-DOTS 30, new SS^+^ PTB	CB-DOT by community volunteers (always neighbors, rather than family members); 2 months of intensive phase); 540 cases in pre-CB-DOTS groups/450 cases in CB-DOTS groups	Administering drugs, noting the treatment cards, monitoring adverse reactions and reminding patients to return to health unit for sputum examination	Successful treatment, cured treatment, completed treatment, death, Failure, transfer out, interrupted treatment	Treatment success among new SS^+^ PTB cases increased from 56% to 74% [RR (95% CI):1.3(1.2, 1.5)] and interrupted treatment decreased from 23% to 1% [RR (95% CI):16.5 (6.1, 44.7)]. There were no significant differences for the rest outcome: Death: 15% pre-CB-DOT vs. 14% post CB-DOT; Cured: 45.3% pre-CB-DOT vs.62.2% post CB-DOT; Completed: 10.9% pre-CB-DOT vs.11.6% post CB-DOT; Failure: 0.9% pre-CB-DOT vs.0% post CB-DOT Transfer out: 5.2% pre-CB-DOT vs.11.2% post CB-DOT; Interrupted: 22.5% pre-CB-DOT vs.1.4% post CB-DOT
Newell, 2006[31]	Cluster RCT	Nepal	≥15 years patients, new SS^+^ PTB	CB-DOT by a female community health volunteer or a village health worker. Family-member DOT was defined as a strategy with drug taking supervised daily by a household member selected by the patient, with drugs provided to the patient’s supervisor every week. Entire standard course of treatment; 549 cases with CB-DOT /358 cases with family-based DOT	The supervisor also traced patients who discontinued treatment	Successful treatment	Community DOT and family-member DOT achieved success rates of 85% and 89%, respectively, but no significant difference [OR (95% CI): 0.67 (0.41, 1.10)].
Sinanovic, 2006[39]	Cohort study	South Africa	no description about ages of patients, new PTB (no description about smear positive or negative)	CB DOT by community health workers (known as ‘treatment supporters’) in the community. No mention period of DOT implementation; 305 cases with public clinics based DOT/ 518 cases with workplace-based DOT / 445 cases with CB-DOTS by CHWs	No mention of additional measures.	Successful treatment, cured treatment, interrupted treatment, failure, transferred out and death	Patients supervised in public clinics generally had lower treatment completion rates than those supervised in the occupational health clinics in the workplace and in the community. Successful treatment rate: 67.2% public clinics based DOT/ 87.1% workplace-based DOT / 72.8% CB-DOT; Cure rate: 63.9% public clinics based DOT vs.77.2% workplace-based DOT vs. 61.1% CB-DOT; Failed: 0.33% public clinics based DOT/0.97% workplace-based DOT vs. 0.90% CB- DOT; Died: 1.64% public clinics based DOT/11.20% workplace-based DOT vs. 3.82% CB- DOT; Interrupted: 14.10% public clinics based DOT/0.00% workplace-based DOT vs. 13.48% CB-DOT; Transfer out: 16.72% public clinics based DOT/0.97% workplace-based DOT vs. 8.99% CB- DOT
Sinanovic, 2003[40]	Cohort study	South Africa	No description about ages of patients, new and retreatment SS^+^ PTB	CB DOT lay-person ‘treatment supporter’; Entire standard course of treatment; NSP: 261 cases with CB-DOT /248 cases with clinic-based DOT/ 16 cases with Workplace-based supervision; Retreatment: 86 cases with CB-DOTS by lay-person ‘treatment supporter’/150 cases with clinic-based DOT/ 4 cases with Workplace-based DOT	No mention of additional measures.	Successful treatment	Community based care had higher successful treatment rate for both new and retreatment TB patients: NSP: 54% clinic-based DOT vs. 80% Community supervision by lay-person ‘treatment supporter’/81% Workplace-based supervision; Retreatment: 49% clinic-based DOT vs. 73% Community supervision by lay-person ‘treatment supporter’/ 75%Workplace-based supervision
Dudley, 2003[41]	Proohort study	South Africa	≥15 years patients, new and retreatment SS^+^ PTB	CB DOT by treatment supporters. Entire standard course of treatment. NSP: 369 cases with CB-DOTS /360 cases with Clinic based DOT. Retreatment: 123 cases with CB-DOTS /203 cases with Clinic based DOT	To note patients’ adherence card and visited patients who did not attend within a week; To recall the patients of default from treatment in 24 hours	Cured treatment, completed treatment, death, failure, transfer out, interrupted treatment	Higher cure rate for the patients with CB-DOTS, treatment success rates were similar. NSP: Cure rate: 72% CB-DOT vs. 46% Clinic based DOT; Complete: 9% CB-DOT vs. 7% Clinic based DOT; Failure:0.3% CB-DOT vs. 0% Clinic based DOT; Died: 1% CB-DOT vs. 3% Clinic based DOT; Interrupted:13% CB-DOT vs. 25% Clinic based DOT;Transfer:5% CB-DOT vs.19% Clinic based DOT; Retreatment: Cure rate: 63% CB-DOT vs. 35% Clinic based DOT; Completed:11% CB-DOT vs.12% Clinic based DOT; Failure:1% CB-DOT vs. 1% Clinic based DOT; Died:2% CB-DOT vs. 10% Clinic based DOT; Interrupted:18% CB-DOT vs. 30% Clinic based DOT; Transfer out:5% CB-DOT vs. 13% Clinic based DOT
Zwarenstein, 2000[42]	RCT	South Africa	≥15 years, new and retreatment PTB	DOT by LHW (took their drugs several times per week at their LHW’s home and under the LHW’s direct supervision); 5 days per week for the first 8 weeks for new patients,12 weeks for re-treatment patients, followed by 3 days per week for the continuation phase; 58 cases with DOT by clinic HW / 44 Self /54 cases with DOT by LHW	To note the adherence card in each visit to the patients, to visited the patients who failed to attend to treatment	Successful treatment, Completed treatment, cured treatment, death, failure, transfer out, interrupted treatment	Successful treatment rates: There were no statistically significant differences across the three supervision options (P = 0.136), but new patients benefit from LHW supervision (compared with clinic nurse and self-supervision, risk difference (95%CI): 24.2% (6, 42.5) and 39.1% (17.8, 60.3) respectively] as do female patients [compared with clinic nurse and self-supervision, risk difference (95%CI): (48.3%(22.8, 73.8) and 32.6% (6.4, 58.7) respectively]; Cured: 41% clinic DOT vs.57% LHW vs.41% Self; Completed: 16% clinic DOT vs.17% LHW vs.18% Self; Failure: 2% clinic DOT vs.6% LHW vs.5% Self; Interrupted: 26% clinic DOT vs.15% LHW vs.25% Self; Transferred out: 16% clinic DOT vs.2% LHW vs.9% Self; Death: 0% clinic DOT vs.4% LHW vs.2% Self.
Singh,2004[43]	Cohort study	India	no description about ages of patients, new SS^+^ PTB	DOT by CV. Entire standard course of treatment;141 cases with DOTS by CVs /476 cases with DOTS by GHWs	To using treatment cards with treatment details during the program, to visit the CVs or patients at least once every 2–3 days when CVs is deviated from standard practice	Successful treatment, cured treatment, default, death, failure, transfer out	There were no significant differences in all outcomes: Cure rate: 70% CVs vs. 75% GHWs [RR (95% CI): 0.95 (0.8, 1.2)]. Successful treatment: 78% CVs vs. 77% GHWs [RR (95% CI): 1.0 (0.8, 1.3)]. Default:15% CVs vs.15% GHWs [RR 1.03(0.7–1.5)]; Death:5% CVs vs.4% GHWs [RR 1.18(0.6–2.3)]; Failure: 2% CVs vs. 4% GHWs [RR 0.57(0.2–1.7)]; Transfer out: 0% CVs vs. 1% g GHWs
Pungrassami,2002[44]	Cohort study	Thailand	Adult and children patients, new and retreatment SS^+^ PTB	CB-DOT by village health volunteers, community Leaders or friends;.Entire standard course of treatment 21 CB-DOT/177 HP DOT / 181 FM/32 SA DOT	No mention of additional measures.	Successful treatment, cured treatment, completed treatment, Death, Failure, Default	There were no significant differences in treatment success between different types of main observers. AOR (95% CI) of treatment non-success were 1.1 (0.3, 4.7). 0.7 (0.2, 3.3), and 0.5(0.2, 1.1) for HP, CM, FM, and SA groups, respectively.
Walley, 2001[45]	RCT	Pakistan	≥15 years patients, new SS^+^ PTB	CB-DOT by a CHW at or near the patient’s home. 2 month of intensive phase; 66 cases with DOT by Health facility staff /104 cases with DOT by CHWs	To record drug-taking form and encouraged patients to complete treatment	Completed treatment, cured treatment, death, failure, transfer out, default	Cure rates: it is higher among TB patients supervised by CHWs (67%) than patients supervised by health facility staff (58%); Completed: 58%Health-facility staff vs. 67% CHWs; Death: 3%Health-facility staff vs. 4% CHWs; Failure: 0%Health-facility staff vs. 1% CHWs; Default: 30%Health-facility staff vs. 25% CHWs; Transfer out: 6%Health-facility staff vs. 0% CHWs
Becx-Bleumink, 2001[46]	Cohort study	Republic of Indonesia	no description about ages of patients, SS^+^ /SS^-^ PTB	CB-DOT by sub-center health workers and village midwives. Entire standard course of treatment; 951 cases with DOT by CBTP /1402 cases with DOT by Non-CBTP	To select tuberculosis suspects, deliver treatment	Successful treatment rate	Treatment success rate (cure and treatment completion): CBTP villages: 90.4%, 89.5% and 93.7% in 1996, 1997, and 1998; Non-CBTP villages: 85.4%, 86.8% and 85.9% in 1996, 1997, and 1998

Notes

DOT refers to direct observation of treatment

NPTB refers to new cases of new cases of smear positive or culture-positive pulmonary tuberculosis

CB refers to community-based

HW refers to health worker

CHW refers to community health workers

LHW refers to lay health workers

NSP refers to new smear positive

NSN refers to new smear negative

NSP refers to new smear positive

CV refers to community volunteers

GHW refers to government health workers

TM refers to traditional hospital-based model of care

CBTP refers to Community based tuberculosis program

The quality assessment indicated that the generation of the allocation sequence for six trials was adequate, but two trials [4,17] lacked information on generation of allocation sequence. Concealment of allocation in three studies [15,16,31] was inadequate, and two studies [4,17] did not provide information. Outcome assessment was blind in only five trials [4,15, 18,31,45]; completeness of follow-up in one trial [17] was assessed as inadequate, two trials did not provide sufficient information to assess this aspect of study quality [42,45] and the rest of the trials were assessed as adequate (S1A Table). All cohort studies met the criteria for ascertainment of exposure, outcome of interest not present at the start of the study, long enough follow-up for outcomes to occur, and complete accounting of follow-up in the cohorts. Only one study [44] did not describe a non-exposed cohort drawn from the same community as the exposed cohort; two studies [19,39] did not describe control of factors (such as severity of disease, health service) that may be associated with treatment outcome. Four studies [19,40,43,46] did not report taking measures to control for additional factors (such as demographic characteristics or socioeconomic factors) which may be associated with treatment outcome; three studies [19,39, 46] did not describe the methods for determining the outcome of treatment. One study scored 6; 2 studies scored 7, the rest 6 studies had scores ≥ 8 (S1B Table).

As for providers of CB-DOT, six studies [4,26,39,42,45–46] involved community health workers for DOT; nine studies had community volunteers, including former TB patients [16], community members [17,19,27,41,43], lay persons [40], village health volunteers or other community leaders [18,44], or friends [44]. One study included both volunteers and community health workers as CB-DOT providers [31]. Two studies also included family members as CB-DOT providers [16, 19]. One RCT by Clarke and colleagues [15] recruited as DOT providers, LHWs who were farm-dwelling peers of adult farm dwellers and had received training on TB.

The period of DOT implementation varied among studies. DOT was implemented in the first 2 months of intensive treatment in four studies [16,17,27,45]. One study [39] did not describe the DOT period, and DOT in one study was conducted 5 days per week for the first 8 weeks for new patients, and 12 weeks for re-treated patients, followed by 3 days per week for the continuation phase [42]. DOT was implemented in the entire standard treatment course in the other 11 studies [4,15, 18–19,26,31,40–41,43,44,46]. Despite the focus of all CB-DOT in monitoring patients while swallowing their anti-TB drugs, all studies included additional measures (such as recording adherence on a Treatment Supporter Card, encouraging patients to complete treatment, noting the adherence card in each visit to the patients, recalling the patients of default from treatment in 24 hours) to improve treatment adherence except in three studies that did not report additional measures [39,40,44] (Table 1). Noticeably, CB-DOT providers, LHWs, also played a mentoring role, visiting the patients, encouraging and monitoring treatment adherence regularly such as in the RCT by Clarke et al. [15].

### Meta-analysis of the impact of CB-DOT on treatment outcome

#### CB-DOT vs. clinic-based DOT

Twelve studies (six RCT and six cohort studies) were included in the meta-analysis on the impact of CB-DOT on TB treatment outcomes compared with clinic-based DOT:

*Twelve studies on all PTB cases*: The heterogeneity test indicated that all studies on successful treatment, cured treatment, treatment default, and death had significant heterogeneity (I^2^ = 87, 88, 42 and 65 respectively) and therefore the random effects model was used for the meta-analysis. However, all studies on completed treatment, treatment failure, and transfer out had no significant heterogeneity (I^2^ = 20, 0, and 38 respectively), and the fixed effects model was used for the meta-analysis. Pooled results demonstrated that CB-DOT promoted successful treatment [pooled RR (95%CI): 1.14 (1.03–1.27)]; reduced treatment default [pooled RR (95%CI): 0.75 (0.58–0.98)] and transfer out [pooled RR (95%CI): 0.39 (0.30–0.50)], but had no effect on curative treatment, treatment completion, treatment default, death, and treatment failure (Table 2).

**Table 2:** Results of meta-analysis of the studies on comparison between CB-DOT and Clinic-based DOT in TB control.

Styles of Meta	No. Of studies	No. of participants	Variance between studies		Pooled RR
			Q(*p*)	(95% CI)	
**All studies on all PTB**					
Successful treatment	12	4915	<0.00001	87	**1.14 [1.03, 1.27]**
Completed Treatment	7	1866	0.28	20	1.24 [0.92, 1.68]
Cured treatment	10	3755	<0.00001	88	1.11 [0.95, 1.29]
Default	10	3308	0.09	42	**0.75 [0.58, 0.98]**
Failed	9	3213	0.8	0	1.37 [0.68, 2.76]
Transfer out	9	3695	0.11	38	**0.39 [0.30, 0.50]**
Death	10	3830	0.002	65	0.70 [0.35, 1.40]
**RCT studies on all PTB**					
Successful treatment	6	1273	0.26	23	**1.11 [1.02, 1.19]**
Completed Treatment	5	751	0.32	14	**1.74 [1.05, 2.90]**
Cured treatment	6	1273	0.4	3	1.07 [0.98, 1.17]
Default	5	826	0.48	0	0.75 [0.52, 1.07]
Failed	6	1348	0.6	0	1.27 [0.55, 2.89]
Transfer out	6	1273	0.14	39	**0.37 [0.23,0.61]**
Death	6	1273	0.11	44	**0.44 [0.26, 0.72]**

*Six RCT trials on all PTB cases*: All studies of all treatment outcome (successful treatment, cured treatment, completed treatment, default, failure, transfer out and death) had no significant heterogeneity. Pooled analysis of six RCTs for all PTB cases demonstrated that CB-DOT promoted successful treatment [pooled RR (95%CI): 1.11 (1.02–1.19)] and treatment completion [pooled RR (95%CI): 1.74 (1.05–1.29)], and reduced mortality [pooled RR (95%CI): 0.44 (0.26–0.72)] and transfer out [pooled RR (95%CI): 0.37 (0.23–0.61)], but had no effect on curative treatment, treatment default, and treatment failure (Table 2).

#### CB-DOT vs. family-based DOT

Three RCTs [4,18,31] and one cohort study [44] were included in the meta-analysis of the effect of CB-DOT on treatment outcome compared with family based-DOT. The heterogeneity test indicated that all studies of five treatment outcome (successful treatment, curative treatment, treatment completion, death, and treatment failure) had no significant heterogeneity. The fixed effects model was therefore, used for the meta-analysis. Pooled results of all studies indicated that there were no significant differences in any treatment outcome between CB-DOT and family-based DOT (Table 3).

**Table 3:** Results of meta-analysis of the studies on comparison between CB-DOT and Family-based DOT, Workplace-based DOT and administered treatment in TB control.

Styles of Meta	No. Of studies	No. of participants	Variance between studies		Pooled RR (95% CI)
			Q(*p*)	I^2^ (%)	
**1. Comparison between CB-DOT and Family-based DOT**					
***Successful treatment***					
All studies on all PTB	4	2880	0.21	33	0.99 [0.95, 1.04]
All cases(RCT)	3	2619	0.17	43	0.99 [0.94,1.03]
***Cured treatment***					
All studies on all PTB	3	1233	0.39	0	1.09 [0.99,1,09]
All cases(RCT)	2	972	0.23	31	1.08 [0.97,1.20]
***Completed treatment***					
All studies on all PTB	3	1233	0.55	0	0.93 [0.75,1.15]
All cases(RCT)	2	972	0.62	0	0.97 [0.791.20,]
***Death***					
All studies on all PTB	3	1973	0.87	0	0.81 [0.63,1.04]
All cases(RCT)	2	1712	0.61	0	0.81 [0.63,1.05]
***Failure***					
All studies on all PTB	3	1772	0.89	0	0.67 [0.13,3.47]
All cases(RCT)	2	1712	0.9	0	0.88 [0.12,6.40]
**2. Comparison of Successful treatment between CB-DOT DOT and Workplace-based DOT**					
All PTB	2	1330	0.19	43	**0.85 [0.79, 0.90]**
New PTB	2	1240	0.2	39	**0.84 [0.79, 0.90]**
**3. Comparison between CB-DOT and Self-administered treatment based on all studies on all PTB)**					
***Successful treatment***	3	534	0.69	0	**1.13 [1.03, 1.24]**
***Cured treatment***	2	225	0.46	0	**1.24 [1.04, 1.48]**
***Completed treatment***	2	225	0.36	0	0.72 [0.32, 1.60]
***Death***	2	225	0.65	0	1.02 [0.29, 3.60]
***Failed treatment***	2	225	0.95	0	1.19 [0.26, 5.50]

#### CB-DOT vs. workplace-based DOT

Only two cohort studies were included in the meta-analysis of the impact of CB-DOT on successful treatment compared with workplace-DOT. These studies had no significant heterogeneity. Pooled results showed that CB-DOT achieved a lower successful treatment rate than workplace-based DOT in all PTB cases [pooled RR (95%CI): 0.85 (0.79–0.90)] (Table 3).

#### CB-DOT vs. self-administered therapy

Three studies (one RCT and two cohort studies) were included in a meta-analysis of the impact of CB-DOT versus self-administered therapy on five treatment outcomes. All studies on successful treatment, curative treatment, treatment completion, death and treatment failure had no significant heterogeneity (I^2^ = 20, 0, and 38 respectively). The pooled results with the fixed effects model demonstrated that CB-DOT promoted successful treatment [pooled RR (95%CI): 1.13 (1.03–1.24)] and curative treatment [pooled RR (95%CI): 1.24 (1.04–1.48)], but had no significant effect on treatment completion [pooled RR (95%CI): 0.72 (0.32–1.60)], death [pooled RR (95%CI): 1.03 (0.29–3.58)] or treatment failure [pooled RR (95%CI): 1.19 (0.26–5.50)] (Table 3).

#### Subgroup analysis

Subgroup analysis was conducted by type of PTB cases (smear-positive PTB, new/retreatment PTB), DOT period (during the intensive treatment period/continuous treatment period) and quality of studies. We found type of PTB cases and DOT period were possible causes of heterogeneity between studies. But we observed no marked influence of type of PTB cases, DOT periods and quality of studies on pooled results (S2A–S2C Table).

## Discussion

DOT was launched by WHO in 1992 [47], and has long been accepted as an effective strategy to promote patient adherence to anti-TB treatment, thus helping to cure most TB cases, to prevent the spread of TB in the community, and to prevent drug-resistant TB [48]. Poor implementation of DOT leads to monotherapy and intermittent treatment, which leads to the emergence of TB drug resistance [48]. CB-DOT is accepted by many countries as a major element of community involvement in TB control [49]. CB-DOT has advantages, particularly in low-to-middle income countries, because costs associated with CB-DOT are typically 40–50% lower than health facility-based care, and the cost-effectiveness of CB-DOT is approximately 50% higher [49]. In response to these findings, more national treatment programs in Africa are now beginning to introduce and expand implementation of CB-DOT as part of routine activities [49]. Before further expansion of CB-DOT, it is necessary to clarify its effect. Thus, this systematic review and meta-analysis updated available evidence on the beneficial effect of CB-DOT on TB control, and suggested that CB-DOT had increased successful treatment rate and completed treatment, and reduced rates of death and transfer out compared with clinic-based DOT. CB-DOT appeared to promote successful treatment and cured treatment compared with self-administered treatment, based on studies with no significant heterogeneity. Workplace-based DOT may have advantage in promoting successful treatment in patients who continue to work during treatment.

Regarding positive treatment outcomes (cured treatment, completed treatment and successful treatment), a review by Lewin et al. [20] provided evidence of moderate quality of the effectiveness of LHWs in improving PTB cure rates [RR (95% CI): 1.22 (1.13–1.31)]. However, the review by Volmink and Garner found no significant difference in clinical outcomes (cure or completion of treatment) between DOT at a clinic versus DOT by a family member or community health worker, or DOT provided by a family member *versus* a community health worker [21]. The current meta-analysis compared CB-DOT with clinic-based DOT, and similarly found that CB-DOT had no advantage in promoting the cure rate in all PTB patients, either based on pooled results of all studies (RCTs and cohort studies) or of RCT studies. However, this meta-analysis found that CB-DOT increased rates of successful treatment in all TB patients (based on all RCT and cohort studies or on only RCTs with the fixed model), and in smear-positive patients (five RCTs fixed model) compared with clinic-based DOT. We also found that CB-DOT promoted completed treatment for all PTB patients (five RCTs, fixed model), and for smear-positive cases (four RCTs, fixed model) compared with clinic-based DOT. In addition, this meta-analysis identified that CB-DOT promoted cured treatment and successful treatment compared with self-administration (one RCT and two cohort studies, fixed model), but workplace DOT had an advantage in successful treatment compared with CB-DOT, based on two cohort studies with no significant heterogeneity.

Regarding negative treatment outcome (default, death, failure, and transfer out), one systematic review and meta-analysis by Toczek et al. [50] demonstrated that the engagement of community health workers as DOT providers and the provision of DOT through outreach treatment were associated with lower default rates for drug-resistant TB. The current meta-analysis identified that CB-DOT significantly reduced death for all PTB patients (six RCTs, fixed model) or for smear-positive PTB patients (five RCTs, fixed model), and the transfer out rate for all cases (nine studies, fixed model) and smear-positive PTB (five RCTs, fixed model) compared with clinic-based DOT.

A previous review [21] concluded that within CB-DOT, comparisons between DOT provided by a family member *versus* a community health worker had similar outcomes. Compared with DOT by family members, our review similarly found that CB-DOT had similar outcomes (successful treatment, cured treatment, competed treatment, death and failure) based on all relevant studies (three RCTs plus one cohort or three RCTs).

Similar to our findings, the review by Karumbi and Garner evaluated DOT [22] compared to self-administered treatment in people on treatment for active TB or on prophylaxis to prevent active disease, and demonstrated little or no difference in cure or treatment completion when DOT was implemented by a family member compared with DOT by community health worker [22]. Another systematic review [23] concluded that DOT was not significantly better than self-administered therapy in preventing microbiologic failure, relapse, or acquired drug resistance. Our review also similar overserved that CB-DOT didn’t improved death rate and failed treatment compared with self-administered therapy.

### Limitations

Our review has some limitations. First, the DOT intervention varied in different studies. Many had additional measures to promote treatment compliance [15–16], while some studies did not mention additional measures [39–40,44]. The differences in the additional measures in different studies were possible reasons for the heterogeneity between studies. However, we could not conduct subgroup analysis or sensitivity analysis because of the limitations in the available data. Second, this review did not cover the impact of CB-DOT on the risk of relapse and time to relapse as well as latent TB infection because few relevant studies were identified. Third, the definition of death by WHO is: “a patient who died from any cause during treatment” [1]. This outcome should focus on death from TB and exclude death from causes other than TB. Finally, the RCTs included in our review did not report which data adhered to the principle of intention-to-treat. Equally, we only included published studies in this review, and this may have led to publication bias [51]. In addition, exclusion of studies with HIV-infected PTB patients is another one limitation.

### Implications

Though a plethora of factors are associated with preventive or curative TB treatment [52], evidence from this meta-analysis shows that CB-DOT, as one key component of community involvement in TB control, can improve TB treatment outcomes. CB-DOT has the potential to contribute to better treatment outcomes, particularly in low-to-middle income countries with high TB burden because of its convenience [19,29,53] and cost-effectiveness [49], and may enable substantial savings in indirect costs associated with clinic-based treatment, including travel costs, child care costs, and loss of earnings. However, CB-DOT needs modification to tailor it to local conditions and perhaps patient preferences. For example: (1) Providers and locations of CB-DOT can be decided by the patients because CB-DOT is patient-centered [4,32,38,53] and community-centered in order to provide flexible and convenient CB-DOT to individual TB patients where they live, work or attend school. (2) Once inexpensive, evidence-based interventions are available in some form, it is important to adapt it to local contexts. Implementation research is use of strategies to adopt, adapt, integrate evidence-based health interventions and policies, and change practice patterns within specific settings [53–55]. Therefore, further implementation research on strategies for implementing CB-DOT in specific community settings would help to provide guidance on how best to integrate evidence-based CB-DOT into the healthcare system [53,55]. (3) The meta-analysis by Kangovi et al. found that CB-DOT programs where providers received financial reward differed possibly from programs without financial reward for providers in TB treatment outcomes [56]. Offering financial incentive to CB-DOT providers are more likely to increase motivation and their effectiveness, but further studies are needed to confirm this hypothesis.

### Conclusions

This systematic review and meta-analysis demonstrated that, as one component of decentralization of TB care from health facilities into the community, “patient centered” and “community centered” CB-DOT did improve treatment outcomes if it tailored to local community conditions. Possibly, it is a promising strategy to scale up CB-DOT in low-to-middle income countries with high TB burden, because it is cost-effective and acceptable. CB-DOT interventions could benefit from further implementation studies to ensure proper tailoring of interventions in line with constraints and resources of the local settings in which they are implemented.

## Supporting Information

S1 PRISMA Checklist PRISMA checklist. (DOC)

S1 Table Quality Assessment of included studies. (DOCX)

S2 Table subgroup analysis. (DOCX)

S1 Text Search strategies. (DOCX)
