# Supplementary material for: Impact of Community-Based DOT on Tuberculosis Treatment Outcomes: A Systematic Review and Meta-Analysis
Source: PLoS One. 2016 Feb 5;11(2):e0147744. doi: 10.1371/journal.pone.0147744 (PMC4744041; doi:10.1371/journal.pone.0147744)
Supplement: S1 Table — (DOCX) [file pone.0147744.s002.docx]

**Table A: Study quality assessment for RCT studies**

| **Study ID** | Allocation sequence  Generation (Adequate, inadequate, unclear) | Allocation  Concealment (Adequate, inadequate, unclear) | Blinding (  assessors) (Adequate, inadequate, unclear) | Completeness of  follow up (Adequate, inadequate, unclear) |
| --- | --- | --- | --- | --- |
| Clarke, 2005[15] | Adequate | Inadequate | Adequate | Adequate |
| Wright, 2004[16] | unclear | unclear | Adequate | Adequate |
| Wandwalo, 2004[17] | Adequate | Inadequate | Inadequate | Adequate |
| Lwilla, 2003[18] | unclear | unclear | Inadequate | Inadequate |
| Kamolratanakul,1999[19] | Adequate | Adequate | Adequate | Adequate |
| Newell, 2006[30] | Adequate | Inadequate | Adequate | Adequate |
| Zwarenstein, 2000[41] | Adequate | Adequate | Inadequate | Unclear |
| Walley, 2001[44] | Adequate | Adequate | Adequate | Unclear |

*Notes:*

*Adequate if steps were taken to ensure the people recording the main outcome of the study were blind to the assigned interventions*

*Inadequate if this was not the case or if there was no mention of attempts to blind the observers.*

*Unclear if not mentioned*

**Table B: Study quality assessment for cohort studies**

| **Study ID** | **Quality assessment criteria** | | | | | | | | | Total Score |
| --- | --- | --- | --- | --- | --- | --- | --- | --- | --- | --- |
|  | Exposed cohort truly representative | Non-exposed cohort drawn from the same community | Right method for ascertainment of exposure | Outcome of interest not present at start of study | Control of factors (severity of disease, health service) may associated with treatment outcome except adherence | Control of any additional factor(demographic characteristics or socio-economic factors) | Quality of outcome assessment | Follow-up long enough for outcomes to occur | Complete accounting for cohorts |  |
| Zvavamwe, 2009[20] | 1 | 1 | 1 | 1 | − | − | − | 1 | 1 | 6 |
| Cavalcante,2007[25] | 1 | 1 | 1 | 1 | 1 | 1 | 1 | 1 | 1 | 9 |
| Adatu, 2003[26] | 1 | 1 | 1 | 1 | 1 | 1 | 1 | 1 | 1 | 9 |
| Sinanovic, 2006[40] | 1 | 1 | 1 | 1 | − | − | 1 | 1 | 1 | 7 |
| Sinanovic, 2003[41] | 1 | 1 | 1 | 1 | 1 | − | 1 | 1 | 1 | 8 |
| Dudley, 2003[40] | 1 | 1 | 1 | 1 | 1 | 1 | 1 | 1 | 1 | 9 |
| Singh,2004[44] | 1 | 1 | 1 | 1 | 1 | − | 1 | 1 | 1 | 8 |
| Pungrassami,2002[45] | 1 | − | 1 | 1 | 1 | 1 | 1 | 1 | 1 | 8 |
| Becx-Bleumink, 2001[47] | 1 | 1 | 1 | 1 | 1 | − | − | 1 | 1 | 7 |

1 indicates the study met the criteria; 0 indicates the study did not meet the criteria; A dash indicates fulfillment of the criteria could not be determined.
