# Supplementary material for: Impact of Community-Based DOT on Tuberculosis Treatment Outcomes: A Systematic Review and Meta-Analysis
Source: PLoS One. 2016 Feb 5;11(2):e0147744. doi: 10.1371/journal.pone.0147744 (PMC4744041; doi:10.1371/journal.pone.0147744)
Supplement: S2 Table — (DOCX) [file pone.0147744.s003.docx]

**Table A Subgroup analysis of comparison between CB-DOT and Clinic-based DOT in TB control.**

| Styles of Meta | No. Of studies | No. of participants | Variance between studies | | Pooled RR |
| --- | --- | --- | --- | --- | --- |
|  |  |  |  |  | (95% CI) |
|  |  |  | Q(*p*) | **I^2^ (%)** |  |
| **Success** | | | | | |
| All studies on all PTB | 12 | 4915 | <0.00001 | 87 | **1.14**  **[1.03, 1.27]** |
| All studies on all PTB *excluded studies only with DOT of first 2months[38,39,43]* | 9 | 4518 | <0.00001 | 86 | **1.12**  **[1.01, 1.24]** |
| All studies on all PTB *excluded studies with retreatment PTB patients[18-19,39-41,43]* | 8 | 2943 | 0.83 | 0 | **1.06 [1.02, 1.11]** |
| All studies on all PTB *excluded studies with without description of “Control of factors” or “Control of any additional factor”[38,42]* | 10 | 3548 | <0.00001 | 89 | **1.16 [1.02, 1.33]** |
| RCT studies with Smear positive PTB | 5 | 1161 | 0.33 | 13 | **1.09 [1.01,1.18]** |
| All studies on all PTB *Excluded RCT with inadequate of quality assessment[18]* | 11 | 4393 | <0.00001 | 89 | 1.15 [1.02, 1.29] |
| RCT studies with All PTB  *Excluded RCT with inadequate of quality assessment[18]* | 4 | 751 | 0.15 | 41 | **1.12 [1.03, 1.21]** |
| **Completed Treatment** | | | | | |
| All studies on all PTB | 7 | 1866 | 0.28 | 20 | 1.24  [0.92, 1.68] |
| All studies on all PTB *excluded studies only with DOT of first 2months[43]* | 6 | 1806 | 0.28 | 21 | 1.28  [0.95, 1.73] |
| RCT studies with Smear positive PTB | 4 | 639 | 0.39 | 1 | **2.22 [1.16,4.23]** |
| **Cured** | | | | | |
| All studies on all PTB | 10 | 3755 | <0.00001 | 88 | 1.11  [0.95, 1.29] |
| All studies on all PTB *Excluded* *studies with without description of “Control of factors” or “Control of any additional factor”[38,42]* | 8 | 2388 | <0.00001 | 87 | 1.16 [0.96, 1.41] |
| All studies on all PTB *Excluded studies only with DOT of first 2months[43]* | 8 | 3695 | <0.00001 | 89 | 1.11  [0.94, 1.31] |
| All studies on all PTB *Excluded* *studies with retreatment PTB patients[18-19,40-41,43]* | 5 | 1948 | 52 | 0 | 0.98 [0.92, 1.04] |
| RCT studies with Smear positive PTB | 2 | 492 | 0.2 | 38 | 1.04 [0.93, 1.15] |
| All studies on all PTB *Excluded RCT with inadequate of quality assessment[18]* | 9 | 3233 | <0.00001 | 89 | 1.11 [0.94, 1.32] |
| RCT studies with All PTB  *Excluded RCT with inadequate of quality assessment[18]* | 5 | 751 | 0.28 | 21 | 1.07 [0.97, 1.17] |
| **Default** | | | | | |
| All studies on all PTB | 10 | 3308 | 0.09 | 42 | **0.75**  **[0.58, 0.98]** |
| All studies on all PTB *Excluded* *studies with without description of “Control of factors” or “Control of any additional factor”[38,42]* | 8 | 1941 | 0.46 | 0 | **0.59 [0.48, 0.73]** |
| All studies on all PTB *Excluded* *studies only with DOT of first 2months[43]* | 9 | 3248 | 0.06 | 49 | 0.76  [0.57, 1.00] |
| All studies on all PTB *Excluded studies with retreatment PTB patients[19,40-41,43]* | 6 | 2023 | 0.72 | 0 | 0.90 [0.71, 1.14] |
| RCT studies with Smear positive PTB | 4 | 714 | 0.4 | 0 | 0.81 [0.54, 1.20] |
| **Failed** | | | | | |
| All studies on all PTB | 9 | 3213 | 0.8 | 0 | 1.37  [0.68, 2.76] |
| All studies on all PTB *Excluded studies with without description of “Control of factors” or “Control of any additional factor”[38,42]* | 7 | 2463 | 0.73 | 0 | 1.24 [0.59, 2.60] |
| RCT studies with Smear positive PTB | 5 | 1236 | 0.56 | 0 | 1.06 [0.43, 2.62] |
| All studies on all PTB *Excluded* *studies only with DOT of first 2months[43]* | 8 | 3153 | 0.78 | 0 | 1.49  [0.72, 3.09] |
| All studies on all PTB *Excluded RCT with inadequate of quality assessment[18]* | 8 | 2691 | 0.79 | 0 | 1.60 [0.73, 3.49] |
| RCT studies with All PTB  *Excluded RCT with inadequate of quality assessment[18]* | 5 | 826 | 0.59 | 0 | 1.57 [0.60, 4.12] |
| **Transfer out** | | | | | |
| All studies on all PTB | 9 | 3695 | 0.11 | 38 | **0.39**  **[0.30, 0.50]** |
| All studies on all PTB *Excluded* *studies with without description of “Control of factors” or “Control of any additional factor”[38,42]* | 7 | 2403 | 0.17 | 34 | **0.32 [0.23, 0.45]** |
| RCT studies with Smear positive PTB | 5 | 1161 | 0.15 | 41 | **0.42 [0.25, 0.70]** |
| All studies on all PTB *Excluded* *studies only with DOT of first 2months[39]* | 8 | 3020 | 0.21 | 27 | **0.33**  **[0.24, 0.45]** |
| All studies on all PTB *Excluded RCT with inadequate of quality assessment[18]* | 8 | 3248 | 0.09 | 44 | **0.40 [0.31, 0.52]** |
| RCT studies with All PTB  *Excluded RCT with inadequate of quality assessment[18]* | 5 | 751 | 0.11 | 46 | **0.45 [0.21, 1.00]** |
| **Death** | | | | | |
| All studies on all PTB | 10 | 3830 | 0.002 | 65 | 0.70  [0.35, 1.40] |
| All studies on all PTB *Excluded* *studies with without description of “Control of factors” or “Control of any additional factor”[38,42]* | 7 | 2463 | 0.06 | 47 | **0.47 [0.23, 0.97]** |
| All studies on all PTB *Excluded* *studies only with DOT of first 2months[43]* | 9 | 3770 | 0.002 | 68 | 0.65  [0.31, 1.35] |
| All studies on all PTB *Excluded studies with retreatment PTB patients[18-19,40-41,43]* | 5 | 2023 | 0.25 | 26 | 1.07 [0.61, 1.87] |
| RCT studies with Smear positive PTB | 5 | 1161 | 0.15 | 40 | **0.39 [0.23,0.66]** |
| All studies on all PTB *Excluded RCT with inadequate of quality assessment[18]* | 9 | 3308 | 0.02 | 55 | 0.85 [0.44, 1.65] |
| RCT studies with All PTB  *Excluded RCT with inadequate of quality assessment[18]* | 9 | 751 | 0 | 59 | 0.74 [0.41, 1.32] |

**Table B Subgroup analysis of comparison between CB-DOT and Self-administration in TB control**

| Styles of Meta | No. Of studies | No.of participants | Variance between studies | | Pooled RR |
| --- | --- | --- | --- | --- | --- |
|  |  |  |  |  | (95% CI) |
|  |  |  | Q(*p*) | **I^2^ (%)** |  |
| **Success** | | | | | |
| All studies on all PTB | 3 | 534 | 0.69 | 0 | **1.13 [1.03, 1.24]** |
| Deleted studies only with DOT of first 2months[43] | 2 | 407 | 0.41 | 0 | 1.14 [1.02, 1.27] |
|  |  |  |  |  |  |

**Table C Subgroup analysis of comparison between CB-DOT and Family-based DOT in TB control.**

| Styles of Meta | No. Of studies | No.of participants | Variance between studies | | Pooled RR |
| --- | --- | --- | --- | --- | --- |
|  |  |  | Q(*p*) | **I^2^ (%)** | (95% CI) |
| **Success** |  |  |  |  |  |
| All studies on all PTB | 4 | 2880 | 0.21 | 33 | 0.99  [0.95, 1.04] |
| Deleted studies only with DOT of first 2months[43] | 3 | 2619 | 0.17 | 43 | 0.99  [0.94, 1.03] |
| **Cured** | | | | | |
| All studies on all PTB | 3 | 1233 | 0.39 | 0 | 1.09 [0.99,1,09] |
| Deleted studies only with DOT of first 2months[43] | 2 | 972 | 0.23 | 31 | 1.08  [0.97, 1.20] |
| **Completed treatment** | | | | | |
| All studies on all PTB | 3 | 1233 | 0.55 | 0 | 0.93 [0.75,1.15] |
| Deleted studies only with DOT of first 2months[43] | 2 | 972 | 0.24 | 28 | 1.23  [0.66, 2.30] |
| **Death** | | | | | |
| All studies on all PTB | 3 | 1973 | 0.87 | 0 | 0.81 [0.63,1.04] |
| Deleted studies only with DOT of first 2months[43] | 2 | 1712 | 0.61 | 0 | 0.81  [0.63, 1.05] |
| **Failure** | | | | | |
| All studies on all PTB | 3 | 1772 | 0.89 | 0 | 0.67 [0.13,3.47] |
| Deleted studies only with DOT of first 2months[43] | 2 | 1812 | 0.9 | 0 | 0.88  [0.12, 6.40] |
