# Supplementary material for: Impact of Community-Based DOT on Tuberculosis Treatment Outcomes: A Systematic Review and Meta-Analysis
Source: PLoS One. 2016 Feb 5;11(2):e0147744. doi: 10.1371/journal.pone.0147744 (PMC4744041; doi:10.1371/journal.pone.0147744)
Supplement: S1 Text — (DOCX) [file pone.0147744.s004.docx]

**Table A: search strategy for Pubmed/Medline**

| Search | Add to builder | Query | Items found |
| --- | --- | --- | --- |
| [#26](http://www.ncbi.nlm.nih.gov/pubmed/advanced) | [Add](http://www.ncbi.nlm.nih.gov/pubmed/advanced) | Search **(((((((((((((((((((((((((Directly observed therapy by Promotora AND tuberculosis treatment outcome)) OR (Directly observed therapy by community health workers AND tuberculosis treatment outcome)) OR (Directly observed therapy by community health workers AND tuberculosis treatment adherence)) OR (Directly observed therapy by primary health workers AND tuberculosis treatment adherence)) OR (Directly observed therapy by lay health workers AND tuberculosis treatment adherence)) OR (Community-Based Directly observed therapy AND tuberculosis treatment outcome)) OR (Community-Based Directly observed therapy AND tuberculosis treatment adherence)) OR (Interventions by community health workers AND tuberculosis treatment outcome)) OR (Interventions by lay health workers AND tuberculosis treatment outcome)) OR (Interventions by primary health workers AND tuberculosis treatment outcome)) OR (Interventions by community health workers AND tuberculosis treatment adherence)) OR (Interventions by lay health workers AND tuberculosis treatment adherence)) OR (Interventions by primary health workers AND tuberculosis treatment adherence)) OR (Community-based intervention AND tuberculosis treatment outcome)) OR (Community-based intervention AND tuberculosis treatment adherence)) OR (Community-based treatment AND tuberculosis treatment outcome)) OR (Community-based treatment AND tuberculosis treatment adherence)) OR (Community-based management AND tuberculosis treatment outcome)) OR (Community-based management AND tuberculosis treatment adherence)) OR (((("Directly Observed Therapy"[Majr]) AND "primary Health Workers"[Mesh]) AND "Tuberculosis "[Mesh]) AND "Patient Compliance"[Mesh])) OR (((("Directly Observed Therapy"[Majr]) AND "lay Health Workers"[Mesh]) AND "Tuberculosis "[Mesh]) AND "Patient Compliance"[Mesh])) OR (((("Directly Observed Therapy"[Majr]) AND "community Health Workers"[Mesh]) AND "Tuberculosis "[Mesh]) AND "Patient Compliance"[Mesh])) OR ((((("Directly Observed Therapy"[Majr]) AND "Community Health Workers"[Mesh]) AND "Tuberculosis "[Mesh]) AND "Treatment Outcome"[Mesh])) OR ((((("Directly Observed Therapy"[Majr]) AND "lay Health Workers"[Mesh]) AND "Tuberculosis "[Mesh]) AND "Treatment Outcome"[Mesh])) OR ((((("Directly Observed Therapy"[Majr]) AND "primary Health Workers"[Mesh]) AND "Tuberculosis "[Mesh]) AND "Treatment Outcome"[Mesh])**  Filters: **Full text; Humans** | [116](http://www.ncbi.nlm.nih.gov/pubmed/?cmd=HistorySearch&querykey=37) |
| [#25](http://www.ncbi.nlm.nih.gov/pubmed/advanced) | [Add](http://www.ncbi.nlm.nih.gov/pubmed/advanced) | Search **(((("Directly Observed Therapy"[Majr]) AND "primary Health Workers"[Mesh]) AND "Tuberculosis "[Mesh]) AND "Treatment Outcome"[Mesh]** | [0](http://www.ncbi.nlm.nih.gov/pubmed/?cmd=HistorySearch&querykey=35) |
| [#24](http://www.ncbi.nlm.nih.gov/pubmed/advanced) | [Add](http://www.ncbi.nlm.nih.gov/pubmed/advanced) | Search **(((("Directly Observed Therapy"[Majr]) AND "lay Health Workers"[Mesh]) AND "Tuberculosis "[Mesh]) AND "Treatment Outcome"[Mesh]** | [0](http://www.ncbi.nlm.nih.gov/pubmed/?cmd=HistorySearch&querykey=33) |
| [#23](http://www.ncbi.nlm.nih.gov/pubmed/advanced) | [Add](http://www.ncbi.nlm.nih.gov/pubmed/advanced) | Search **(((("Directly Observed Therapy"[Majr]) AND "Community Health Workers"[Mesh]) AND "Tuberculosis "[Mesh]) AND "Treatment Outcome"[Mesh]** | [3](http://www.ncbi.nlm.nih.gov/pubmed/?cmd=HistorySearch&querykey=32) |
| [#22](http://www.ncbi.nlm.nih.gov/pubmed/advanced) | [Add](http://www.ncbi.nlm.nih.gov/pubmed/advanced) | Search **((("Directly Observed Therapy"[Majr]) AND "community Health Workers"[Mesh]) AND "Tuberculosis "[Mesh]) AND "Patient Compliance"[Mesh]** | [2](http://www.ncbi.nlm.nih.gov/pubmed/?cmd=HistorySearch&querykey=31) |
| [#21](http://www.ncbi.nlm.nih.gov/pubmed/advanced) | [Add](http://www.ncbi.nlm.nih.gov/pubmed/advanced) | Search **((("Directly Observed Therapy"[Majr]) AND "lay Health Workers"[Mesh]) AND "Tuberculosis "[Mesh]) AND "Patient Compliance"[Mesh]** | [0](http://www.ncbi.nlm.nih.gov/pubmed/?cmd=HistorySearch&querykey=29) |
| [#20](http://www.ncbi.nlm.nih.gov/pubmed/advanced) | [Add](http://www.ncbi.nlm.nih.gov/pubmed/advanced) | Search **((("Directly Observed Therapy"[Majr]) AND "primary Health Workers"[Mesh]) AND "Tuberculosis "[Mesh]) AND "Patient Compliance"[Mesh]** | [0](http://www.ncbi.nlm.nih.gov/pubmed/?cmd=HistorySearch&querykey=27) |
| [#19](http://www.ncbi.nlm.nih.gov/pubmed/advanced) | [Add](http://www.ncbi.nlm.nih.gov/pubmed/advanced) | Search **Community-based management AND tuberculosis treatment adherence** | [19](http://www.ncbi.nlm.nih.gov/pubmed/?cmd=HistorySearch&querykey=20) |
| [#18](http://www.ncbi.nlm.nih.gov/pubmed/advanced) | [Add](http://www.ncbi.nlm.nih.gov/pubmed/advanced) | Search **Community-based management AND tuberculosis treatment outcome** | [32](http://www.ncbi.nlm.nih.gov/pubmed/?cmd=HistorySearch&querykey=19) |
| [#17](http://www.ncbi.nlm.nih.gov/pubmed/advanced) | [Add](http://www.ncbi.nlm.nih.gov/pubmed/advanced) | Search **Community-based treatment AND tuberculosis treatment adherence** | [36](http://www.ncbi.nlm.nih.gov/pubmed/?cmd=HistorySearch&querykey=18) |
| [#16](http://www.ncbi.nlm.nih.gov/pubmed/advanced) | [Add](http://www.ncbi.nlm.nih.gov/pubmed/advanced) | Search **Community-based treatment AND tuberculosis treatment outcome** | [76](http://www.ncbi.nlm.nih.gov/pubmed/?cmd=HistorySearch&querykey=17) |
| [#15](http://www.ncbi.nlm.nih.gov/pubmed/advanced) | [Add](http://www.ncbi.nlm.nih.gov/pubmed/advanced) | Search **Community-based intervention AND tuberculosis treatment adherence** | [7](http://www.ncbi.nlm.nih.gov/pubmed/?cmd=HistorySearch&querykey=16) |
| [#14](http://www.ncbi.nlm.nih.gov/pubmed/advanced) | [Add](http://www.ncbi.nlm.nih.gov/pubmed/advanced) | Search **Community-based intervention AND tuberculosis treatment outcome** | [8](http://www.ncbi.nlm.nih.gov/pubmed/?cmd=HistorySearch&querykey=15) |
| [#13](http://www.ncbi.nlm.nih.gov/pubmed/advanced) | [Add](http://www.ncbi.nlm.nih.gov/pubmed/advanced) | Search **Interventions by primary health workers AND tuberculosis treatment adherence** | [6](http://www.ncbi.nlm.nih.gov/pubmed/?cmd=HistorySearch&querykey=14) |
| [#12](http://www.ncbi.nlm.nih.gov/pubmed/advanced) | [Add](http://www.ncbi.nlm.nih.gov/pubmed/advanced) | Search **Interventions by lay health workers AND tuberculosis treatment adherence** | [6](http://www.ncbi.nlm.nih.gov/pubmed/?cmd=HistorySearch&querykey=13) |
| [#11](http://www.ncbi.nlm.nih.gov/pubmed/advanced) | [Add](http://www.ncbi.nlm.nih.gov/pubmed/advanced) | Search **Interventions by community health workers AND tuberculosis treatment adherence** | [8](http://www.ncbi.nlm.nih.gov/pubmed/?cmd=HistorySearch&querykey=12) |
| [#10](http://www.ncbi.nlm.nih.gov/pubmed/advanced) | [Add](http://www.ncbi.nlm.nih.gov/pubmed/advanced) | Search **Interventions by primary health workers AND tuberculosis treatment outcome** | [2](http://www.ncbi.nlm.nih.gov/pubmed/?cmd=HistorySearch&querykey=11) |
| [#9](http://www.ncbi.nlm.nih.gov/pubmed/advanced) | [Add](http://www.ncbi.nlm.nih.gov/pubmed/advanced) | Search **Interventions by lay health workers AND tuberculosis treatment outcome** | [3](http://www.ncbi.nlm.nih.gov/pubmed/?cmd=HistorySearch&querykey=10) |
| [#8](http://www.ncbi.nlm.nih.gov/pubmed/advanced) | [Add](http://www.ncbi.nlm.nih.gov/pubmed/advanced) | Search **Interventions by community health workers AND tuberculosis treatment outcome** | [8](http://www.ncbi.nlm.nih.gov/pubmed/?cmd=HistorySearch&querykey=9) |
| [#7](http://www.ncbi.nlm.nih.gov/pubmed/advanced) | [Add](http://www.ncbi.nlm.nih.gov/pubmed/advanced) | Search **Community-Based Directly observed therapy AND tuberculosis treatment adherence** | [18](http://www.ncbi.nlm.nih.gov/pubmed/?cmd=HistorySearch&querykey=8) |
| [#6](http://www.ncbi.nlm.nih.gov/pubmed/advanced) | [Add](http://www.ncbi.nlm.nih.gov/pubmed/advanced) | Search **Community-Based Directly observed therapy AND tuberculosis treatment outcome** | [42](http://www.ncbi.nlm.nih.gov/pubmed/?cmd=HistorySearch&querykey=7) |
| [#5](http://www.ncbi.nlm.nih.gov/pubmed/advanced) | [Add](http://www.ncbi.nlm.nih.gov/pubmed/advanced) | Search **Directly observed therapy by lay health workers AND tuberculosis treatment adherence** | [3](http://www.ncbi.nlm.nih.gov/pubmed/?cmd=HistorySearch&querykey=6) |
| [#4](http://www.ncbi.nlm.nih.gov/pubmed/advanced) | [Add](http://www.ncbi.nlm.nih.gov/pubmed/advanced) | Search **Directly observed therapy by primary health workers AND tuberculosis treatment adherence** | [5](http://www.ncbi.nlm.nih.gov/pubmed/?cmd=HistorySearch&querykey=5) |
| [#3](http://www.ncbi.nlm.nih.gov/pubmed/advanced) | [Add](http://www.ncbi.nlm.nih.gov/pubmed/advanced) | Search **Directly observed therapy by community health workers AND tuberculosis treatment adherence** | [18](http://www.ncbi.nlm.nih.gov/pubmed/?cmd=HistorySearch&querykey=4) |
| [#2](http://www.ncbi.nlm.nih.gov/pubmed/advanced) | [Add](http://www.ncbi.nlm.nih.gov/pubmed/advanced) | Search **Directly observed therapy by community health workers AND tuberculosis treatment outcome** | [32](http://www.ncbi.nlm.nih.gov/pubmed/?cmd=HistorySearch&querykey=3) |
| [#1](http://www.ncbi.nlm.nih.gov/pubmed/advanced) | [Add](http://www.ncbi.nlm.nih.gov/pubmed/advanced) | Search **Directly observed therapy by Promotora AND tuberculosis treatment outcome** | [0](http://www.ncbi.nlm.nih.gov/pubmed/?cmd=HistorySearch&querykey=1) |

**Table B: search strategy for** EBSCO

| [Search ID#](javascript:__doPostBack('ctl00$ctl00$FindField$FindField$historyControl$ReorderHistoryLink','')) | **Search Terms** | **Search Options** | **Actions** | |
| --- | --- | --- | --- | --- |
|  | S13 | (S1 OR S2 OR S3 OR S4 OR S5 OR S6 OR S7 OR S8 OR S9 OR S10 OR S11 OR S12) | **Search modes** - Boolean/Phrase | [**View Results**](javascript:__doPostBack('ctl00$ctl00$FindField$FindField$historyControl$HistoryRepeater$ctl00$linkResults','')) (6) |
|  | S12 | pulmonary tuberculosis AND community | **Limiters** - Linked Full Text; Publication Type: All Journals; Population Group: Human; Research Article; Exclude MEDLINE records; Human; Publication Type: Academic Journal  **Search modes** - Boolean/Phrase | [**View Results**](javascript:__doPostBack('ctl00$ctl00$FindField$FindField$historyControl$HistoryRepeater$ctl00$linkResults','')) (6) |
|  | S11 | pulmonary tuberculosis AND primary health | **Limiters** - Linked Full Text; Publication Type: All Journals; Population Group: Human; Research Article; Exclude MEDLINE records; Human; Publication Type: Academic Journal  **Search modes** - Boolean/Phrase | [**View Results**](javascript:__doPostBack('ctl00$ctl00$FindField$FindField$historyControl$HistoryRepeater$ctl00$linkResults','')) (1) |
|  | S10 | pulmonary tuberculosis AND community health | **Limiters** - Linked Full Text; Publication Type: All Journals; Population Group: Human; Research Article; Exclude MEDLINE records; Human; Publication Type: Academic Journal  **Search modes** - Boolean/Phrase | [**View Results**](javascript:__doPostBack('ctl00$ctl00$FindField$FindField$historyControl$HistoryRepeater$ctl00$linkResults','')) (1) |
|  | S9 | pulmonary tuberculosis AND treatment adherence AND community | **Limiters** - Linked Full Text; Publication Type: All Journals; Population Group: Human; Research Article; Exclude MEDLINE records; Human; Publication Type: Academic Journal  **Search modes** - Boolean/Phrase | [**View Results**](javascript:__doPostBack('ctl00$ctl00$FindField$FindField$historyControl$HistoryRepeater$ctl00$linkResults','')) (2) |
|  | S8 | pulmonary tuberculosis AND treatment compliace AND community | **Limiters** - Linked Full Text; Publication Type: All Journals; Population Group: Human; Research Article; Exclude MEDLINE records; Human; Publication Type: Academic Journal  **Search modes** - Boolean/Phrase | [**View Results**](javascript:__doPostBack('ctl00$ctl00$FindField$FindField$historyControl$HistoryRepeater$ctl00$linkResults','')) (0) |
|  | S7 | pulmonary tuberculosis AND Directly observed therapy AND primary health care | **Limiters** - Linked Full Text; Publication Type: All Journals; Population Group: Human; Research Article; Exclude MEDLINE records; Human; Publication Type: Academic Journal  **Search modes** - Boolean/Phrase | [**View Results**](javascript:__doPostBack('ctl00$ctl00$FindField$FindField$historyControl$HistoryRepeater$ctl00$linkResults','')) (0) |
|  | S6 | pulmonary tuberculosis AND Directly observed therapy AND community health care | **Limiters** - Linked Full Text; Publication Type: All Journals; Population Group: Human; Research Article; Exclude MEDLINE records; Human; Publication Type: Academic Journal  **Search modes** - Boolean/Phrase | [**View Results**](javascript:__doPostBack('ctl00$ctl00$FindField$FindField$historyControl$HistoryRepeater$ctl00$linkResults','')) (0) |
|  | S5 | pulmonary tuberculosis AND Directly observed therapy AND primary health worker | **Limiters** - Linked Full Text; Publication Type: All Journals; Population Group: Human; Research Article; Exclude MEDLINE records; Human; Publication Type: Academic Journal  **Search modes** - Boolean/Phrase | [**View Results**](javascript:__doPostBack('ctl00$ctl00$FindField$FindField$historyControl$HistoryRepeater$ctl00$linkResults','')) (0) |
|  | S4 | pulmonary tuberculosis AND Directly observed therapy AND lay health worker | **Limiters** - Linked Full Text; Publication Type: All Journals; Population Group: Human; Research Article; Exclude MEDLINE records; Human; Publication Type: Academic Journal  **Search modes** - Boolean/Phrase | [**View Results**](javascript:__doPostBack('ctl00$ctl00$FindField$FindField$historyControl$HistoryRepeater$ctl00$linkResults','')) (0) |
|  | S3 | pulmonary tuberculosis AND Directly observed therapy AND community health worker | **Limiters** - Linked Full Text; Publication Type: All Journals; Population Group: Human; Research Article; Exclude MEDLINE records; Human; Publication Type: Academic Journal  **Search modes** - Boolean/Phrase | [**View Results**](javascript:__doPostBack('ctl00$ctl00$FindField$FindField$historyControl$HistoryRepeater$ctl00$linkResults','')) (0) |
|  | S2 | pulmonary tuberculosis AND Directly observed therapy | **Limiters** - Linked Full Text; Publication Type: All Journals; Population Group: Human; Research Article; Exclude MEDLINE records; Human; Publication Type: Academic Journal  **Search modes** - Boolean/Phrase | [**View Results**](javascript:__doPostBack('ctl00$ctl00$FindField$FindField$historyControl$HistoryRepeater$ctl00$linkResults','')) (0) |
|  | S1 | ( pulmonary tuberculosis AND Directly observed therapy ) AND community health worker | **Limiters** - Linked Full Text; Publication Type: All Journals; Population Group: Human; Research Article; Exclude MEDLINE records; Human; Publication Type: Academic Journal  **Search modes** - Boolean/Phrase | [**View Results**](javascript:__doPostBack('ctl00$ctl00$FindField$FindField$historyControl$HistoryRepeater$ctl00$linkResults','')) (0) |

**Table C: search strategy for** EMBAS

| **#10** | #1 OR #2 OR #3 OR #4 OR #5 OR #6 OR #7 OR #8 OR #9 | 53 |
| --- | --- | --- |
| **#9** | **directly AND observed AND ('therapy'/exp OR therapy) AND andpulmonary AND ('tuberculosis'/exp OR tuberculosis) ANDtreatment AND outcome AND ('community'/exp OR community) AND ('health'/exp OR health) AND ('worker'/exp OR worker) AND [article]/lim AND [humans]/lim AND [embase]/lim** | 0 |
| **#8** | **directly AND observed AND ('therapy'/exp OR therapy) AND andpulmonary AND ('tuberculosis'/exp OR tuberculosis) ANDtreatment AND outcome AND lay AND ('health'/exp OR health) AND ('worker'/exp OR worker)** | 0 |
| **#7** | directly AND observed AND therapy AND tuberculosis AND treatment AND outcome AND promotora | 0 |
| **#6** | directly AND observed AND ('therapy'/exp OR therapy) AND pulmonary AND ('tuberculosis'/exp OR tuberculosis) AND treat ANDoutcome AND [article]/lim AND [humans]/lim AND [embase]/lim | 4 |
| **#5** | pulmonary AND ('tuberculosis'/exp OR tuberculosis) AND directly AND observed AND ('therapy'/exp OR therapy) AND primaryAND ('health'/exp OR health) AND care AND provider AND [article]/lim AND [humans]/lim AND [embase]/lim | 0 |
| **#4** | **pulmonary AND ('tuberculosis'/exp OR tuberculosis) AND directly AND observed AND ('therapy'/exp OR therapy) AND lay AND ('health'/exp OR health) AND ('worker'/exp OR worker) AND [article]/lim AND [humans]/lim AND [embase]/lim** | 1 |
| **#3** | pulmonary AND ('tuberculosis'/exp OR tuberculosis) AND directly AND observed AND ('therapy'/exp OR therapy) AND ('community'/exp OR community) AND ('health'/exp OR health) AND [article]/lim AND [humans]/lim AND [embase]/lim | 40 |
| **#2** | **pulmonary** AND (**'tuberculosis'**/exp OR **tuberculosis**) AND **directly** AND **observed** AND (**'therapy'**/exp OR **therapy**) AND **primary**AND (**'health'**/exp OR **health**) AND **care** AND [article]/lim AND [humans]/lim AND [embase]/lim | 13 |
| **#1** | **pulmonary AND ('tuberculosis'/exp OR tuberculosis) AND directly AND observed AND ('therapy'/exp OR therapy) AND ('community'/exp OR community) AND ('health'/exp OR health) AND ('worker'/exp OR worker) AND [article]/lim AND [humans]/lim AND [embase]/lim** | 2 |
